# Children’s Environmental Health: Homes of Influence

**DOI:** 10.1289/EHP749

**Published:** 2016-12-01

**Authors:** Brenda Afzal, Nsedu Obot Witherspoon, Kristie Trousdale

**Affiliations:** Children’s Environmental Health Network (CEHN), Washington, DC, USA

## Abstract

Over the past two decades, diverse stakeholder groups, representing various disciplines and perspectives (e.g., federal, state and local policy makers; nonprofit organizations; health professionals; and industry), have devoted considerable resources, expertise, and influence toward efforts that wittingly and unwittingly affect children’s environmental health. In this article, we refer to these groups as “homes of influence,” and we summarize the wide reach and potential impacts of these homes on childhood asthma, as one example that illustrates how these varied groups impact childhood health outcomes. We posit that diverse homes of influence can be most successful in effecting positive change when they understand and acknowledge their respective influences and work together to develop informed, preventive initiatives under the framework of recommendations called, “A Blueprint for Protecting Children’s Environmental Health: An Urgent Call to Action.” This published resource was developed by a panel of thought leaders and experts in the field of children’s environmental health to guide cross-sector collaboration efforts to proactively protect the environmental health of all children.

## A Blueprint for Protecting Children’s Environmental Health: An Urgent Call to Action

Over the past three years, the Children’s Environmental Health Network (CEHN), a national multi-disciplinary nonprofit organization with the primary mission to protect all children from environmental hazards, has led efforts to assess the field of children’s environmental health (CEH) for the benefit of more effective and efficient actions. CEHN has worked with numerous stakeholder groups with the common goal of providing safe and healthy environments for children. Stemming from these efforts, and in collaboration with leaders in the field, we have developed a resource report—“A Blueprint for Protecting Children’s Environmental Health: An Urgent Call to Action” (CEHN 2015a)—with a suite of recommendations that presents the case for identifying, acknowledging, and working within the many homes of influence that affect the field of children’s environmental health and influence children’s environments, whether positively or negatively.

In this article, the phrase “homes of influence” refers to individuals, organizations, government entities, coalitions, and others (e.g., grassroots organizations and stakeholders across multiple sectors) that can influence opinions and behavior in groups or individuals through the use of power, leadership, and authority, both legitimate and conferred. These homes can and should be united into a system that we refer to here as a neighborhood of influence. Far too many of these homes do their good work in isolation, which can lead to duplication of effort, loss of valuable resources, and ultimately, failure to achieve long-term success. The homes of influence in CEH represent varied fields and perspectives, including health professionals; nonprofit advocacy organizations; community stakeholders with particular environmental and health concerns; federal, state, and municipal governments and policy makers; the research community; educators; industry; and funders from both government entities and private foundations. In this rapidly changing world, with its growing population and ever-evolving marketplace, it is crucial for those working to protect CEH to know all the players, the power brokers, and the homes of influence that will make a difference in CEH in the years ahead.

### Using Asthma as an Example of the Need for Action

We use asthma to illustrate how multiple sectors or homes influence or contribute to, positively or negatively, the burden of childhood asthma via environmental factors. Asthma, a chronic respiratory illness, is one of the most common chronic disorders in childhood that currently affects an estimated 6.3 million children < 18 years old in the United States (CDC 2014a). It is a leading cause of missed school days and hospital visits for children. Although the underlying causes of this complex disease are still not fully understood, researchers agree that gene–environment interactions are clearly at play (McLeish and Turner 2007; Mukherjee and Zhang 2011; Murdoch and Lloyd 2010). Besides playing a role in the development of new-onset asthma (Chen et al. 2015), some environmental factors, such as air pollution, can affect the frequency and the severity of the symptoms that manifest in children already diagnosed. The homes of influence for understanding environmental contributions to asthma and the actions needed to prevent or mitigate them comprise almost all significant areas of the child’s environment.

In the United States, federally funded research on asthma, its causes, exacerbations, treatments, and most importantly prevention, has been led by three institutes within the National Institutes of Health—the National Heart, Lung, and Blood Institute (NHLBI), the National Institute of Environmental Health Sciences (NIEHS), and the National Institute of Allergy and Infectious Diseases (NIAID)—and the U.S. Environmental Protection Agency (EPA). Together, these federal institutes and their programs (e.g., NHLBI’s National Asthma Education and Prevention Program and NIAID’s Inner-city Asthma Consortium) contribute to our understanding of the causes of asthma; develop ways to better manage this condition; and assess intervention efforts, which translate scientific discoveries into clinical practice. Significant funding also comes from private entities. The American Asthma Foundation Research Program is the largest private funder of asthma research that has awarded more than $100 million to scientists since 2000 (AAF 2016). Biopharmaceutical research companies, in their quest for new medicines to treat asthma, spend on average, more than $1 billion on each new drug (PhRMA 2012). Although billions of dollars are invested in the treatment of asthma, including the development, effectiveness, and safety of pharmaceuticals, providing benefits to individuals with asthma, much more attention to primary prevention is needed (CDC 2013). This goal is highlighted as one of four major actions being advanced by U.S. federal partners in the “President’s Task Force on Environmental Health Risks and Safety Risks to Children: Coordinated Federal Action Plan to Reduce Racial and Ethnic Asthma Disparities” (U.S. EPA 2012). Because prevention research dollars may be insufficient to address this need in today’s challenging economy and political environment, new sources of funding will be needed.

Public health agencies and departments of health at the federal, state, and local levels can also participate in asthma research, as well as prevention, surveillance and tracking, and intervention and asthma management services. The Centers for Disease Control and Prevention’s (CDC) National Asthma Control Program funds initiatives at state and local levels throughout the country in surveillance, education, training, and awareness. Between 1999, when the Program was created, and 2013, asthma deaths decreased by 27%: The return on investment was valued at $71 saved on asthma-related expenses for each dollar spent on national and local services (CDC 2013). On the other hand, recent budget cuts to the National Asthma Control Program have resulted in less funding to fewer states for these important services. Only 23 states now receive funding, down from 34 (CDC 2013, 2014c; NCHH 2016).

Health care professionals, such as the American Academy of Pediatrics (AAP), the National Association of School Nurses (NASN), the American Academy of Allergy, Asthma, and Immunology (AAAAI), the National Medical Association, the American Public Health Association, and others, have significant ongoing efforts underway to educate their membership, policy makers, the media, and the general public about the causes, triggers, treatments, and prevention of asthma (Li 2014; Cohn and Martin 2009; NASN 2015). Health care professionals are a trusted and influential, but underutilized, advocacy resource. While it is assumed that these organizations will continue to engage in education and advocacy efforts, additional health professional organizations should be encouraged to share best practice materials with their members who in turn should be encouraged to join the efforts to educate and engage policy makers on this important issue. There will be continued opportunities to build health care professional champions for asthma prevention and children’s environmental health overall.

Environmental asthma triggers and exposures occur indoors and out, across places such as homes, schools, and childcare facilities, where children spend the bulk of their time. The built environment can greatly influence childhood asthma (Perdue et al. 2003). Children living or attending school in highly urban areas near polluting industry or major roadways can be exposed to high levels of particulate matter or other emitted respiratory irritants. Ground-level ozone and other air pollutants can trigger asthma symptoms and lead to asthma flare-ups. Children in sprawling suburban areas can also be exposed to significant traffic pollution. Therefore, local zoning boards, transportation departments, and planning departments continue to be an important sphere of influence, and thus are best informed from increased participation of public health professionals and advocates.

Children spend a significant amount of time indoors where the air may be as polluted as or even more polluted than outdoor air (U.S. EPA 2016). Thus, in addition to zoning and land-use decisions, substandard housing can contribute significantly to the childhood asthma burden because of the presence of dampness and mold, pest infestations, poor ventilation, and other factors affecting indoor air quality (Northridge et al. 2010). The National Center for Healthy Housing (NCHH; http://www.nchh.org/) and the Green and Healthy Homes Initiative (http://www.greenandhealthyhomes.org/) are two influential nonprofit organizations that focus on creating safer, healthier housing through advocacy, education, training, research, and improved policy. Other players in the housing arena include state and local housing authorities funded by the U.S. Department of Housing and Urban Development, private developers, the American Association of Code Enforcement, the U.S. Green Building Council, and more.

Outside of the home, young children spend the majority of their time in schools or childcare facilities, making these settings, and all relevant players influencing the environmental health of these settings, important with regard to the prevention and management of childhood asthma. The condition of the nation’s school facilities is a significant concern. Funding, especially in underserved communities, is often limited; many facilities are poorly maintained; and some are located near industrial plants or hazardous waste sites. Overcrowded conditions in many schools compromise ventilation systems, and maintenance practices can contribute to poor indoor air quality. In addition, despite recent efforts to replace or retrofit old diesel-fueled school buses and reduce or eliminate idling times, there are still approximately 250,000 old diesel buses in use in the United States (MacMillan 2016). Children in the United States are mandated to attend school yet have no recourse or support services, such as provided to adults by the Occupational Safety and Health Administration, when exposed to environmental hazards in schools or childcare facilities. There is no nationally coordinated effort or policies to track, address, or prevent school and early-learning exposures, which is a significant gap in the protection of children’s health (HSN 2015). The U.S. Department of Education, state and local boards of education, and school districts are clear stakeholders with regard to this issue, because asthma accounts for approximately 14 million missed schools days each year (CDC 2015).

The youngest and most vulnerable of our nation’s children are not yet in schools. Approximately six million children in the United States receive early-care and learning experiences outside of the home (Laughlin 2013). Licensed childcare programs are regulated via a patchwork of state and local agencies including childcare licensing offices, state or local departments of health, and departments of environment. Many factors pertinent to healthy indoor air quality in childcare facilities are not currently regulated by these agencies (Seltenrich 2013). Some states have been incorporating environmental health standards into their voluntary Quality Rating Improvement System (QRIS; https://qrisguide.acf.hhs.gov/) programs, which provide recognition to childcare programs that go above and beyond licensing requirements in providing quality environments for child learning and development. In addition, accreditation organizations wield significant influence in the childcare industry. The two largest organizations, the National Association for the Education of Young Children (www.naeyc.org) and the Association for Early Learning Leaders (http://www.earlylearningleaders.org/), are both in the process of incorporating environmental health criteria and standards into their voluntary accreditation requirements (CEHN 2015b, 2015c). The Agency for Toxic Substances and Disease Registry (ATSDR) is planning to release guidelines for safe siting of childcare facilities to assist state and local authorities and childcare program directors in reducing children’s potential exposures to air pollution from establishing childcare facilities on or adjacent to hazardous sites (Somers and Ulirsch 2016).

Model voluntary recognition programs and best practice guidelines can account for some air quality improvements in settings where children spend a great deal of their time, and often they can serve as helpful models for eventual policy and regulatory change. However, many in communities with specific health problems derived from the environment may not be aware of, nor have access to, these resources or the support needed to undertake the best practice recommendations. Proactive, evidence-based policies and regulations (to reduce or prevent pollution emissions and the introduction of harmful chemicals into consumer products) are better suited to protect all children.

Federal regulatory efforts to protect outdoor air quality have been authorized since the enactment of the Clean Air Act of 1970 (1970). In general, the more specific enforcement mechanisms for such efforts may be prescribed by the executive branch, but still need support and leadership of legislative champions from both sides of the aisle. Nonetheless, implementation is left to regulatory agencies such as the U.S. EPA that set and enforce standards for ambient air pollutants at both the state and federal level. The U.S. EPA’s mission is to protect human health and the environment. However, the agency’s authority is determined by provisions of environmental laws. This situation constrains the extent to which the U.S. EPA can accomplish its very broad mission. For example, in 2001 a U.S. Supreme Court decision (Whitman *v.* American Trucking Associations, Inc.) interpreted the Clean Air Act’s mandate to the U.S. EPA to preclude economic cost considerations in setting National Ambient Air Quality Standards, such as for ozone; rather, decisions must be based only on the scientific evidence pertaining to human and environmental health effects. However, under Executive Orders 12866 and 13563 (The President 1993, 2011), the agency is required to perform, and submit for review, cost-benefit analyses for proposed rule implementation in order to justify the costs of regulation. According to a professor of American politics, R.S. Melnick, “The risks, costs, and benefits under scrutiny are usually difficult to estimate with precision” (NRC 1990). In the case of air pollution, the true costs of the existing childhood asthma burden and other respiratory health conditions that would be reduced with the promulgation of a new or tighter rule are difficult to ascertain due to numerous intangible and invaluable factors, such as children’s quality of life, that may not be factored into the analysis. Furthermore, industries that contribute to air pollution may oppose regulations that require them to assume the associated costs of pollution reduction.

The private sector—particularly industry and entities such as fossil fuel energy companies and the automobile industry—wields significant influence over ambient air pollution rules and enforcement of those rules (Greene et al. 2011). In one study, Wagner et al. (2011) examined participation and influence of special interest groups on U.S. EPA regulations at several key stages of the rulemaking process. Specifically, the study reviewed the promulgation of emission standards for toxic air pollutants for more than 100 major industries. The results revealed high levels of participation and stark imbalances among participating groups at each of these stages, with industry having a significant advantage over public interest groups. Common arguments against stiffer regulations are that greater costs to industry imposed by stronger regulations *a*) threaten the industry’s responsibility to their investors, *b*) result in fewer jobs in the affected communities, and *c*) increase costs to consumers. These arguments often fail to consider the fact that many consumers may already be paying higher health care costs due to under-regulated pollution. The Natural Resources Defense Council offered an example of industry’s impact on regulatory issues in a 2012 report: “As an example of the problem, despite a 10-year effort to ban asbestos and overwhelming evidence that it is deadly, the EPA lost an industry challenge in court and was not allowed to ban existing uses. As a result, although companies no longer mine asbestos in the United States, we continue to import products containing asbestos and ten thousand people die each year from past and on-going exposures” (Sass and Rosenberg 2011).

Beyond the rulemaking process, enforcement may be a weak link in efforts to mitigate asthma risks. For example, the American Lung Association’s (ALA) 2016 report, “State of the Air®,” noted that despite the existence of federal air quality standards, more than half of all Americans reside in counties that have ozone and particulate matter levels that exceed their respective standards (ALA 2016). A U.S. EPA report by the Office of Inspector General (OIG) indicated that the “EPA does not administer a consistent national enforcement program” (U.S. EPA 2011). This report further stated that “Despite efforts by the Office of Enforcement and Compliance Assurance (OECA) and the EPA regions to improve state enforcement performance, state enforcement programs frequently do not meet national goals, and states do not always take necessary enforcement actions” (U.S. EPA 2011). At the time of this report, the OIG found that state enforcement programs are underperforming and that the U.S. EPA data indicate high levels of noncompliance and low levels of enforcement.

Industry is influential with regard to indoor air quality as well as with regard to polluting emissions and ambient air. Not only do outdoor pollutants pervade indoors, but many manufactured interior furnishings and consumer products contribute respiratory irritants into the air. Third-party certifiers of cleaning products, such as the U.S. EPA’s Safer Choice program, GreenSeal™, and UL Ecologo™, and certifiers of furnishings such as GREENGUARD, help consumers and large purchasing agents identify which products contain fewer chemicals of concern. In June of 2016, President Obama signed into law the Frank R. Lautenberg Chemical Safety for the 21st Century Act (2016), which amends the Toxic Substances Control Act (TSCA) of 1976 (1976), the primary federal law that addresses the management and safety of chemicals. The new safety assessments of chemicals under this TSCA reform law will require a health-based risk evaluation and environmental safety standard, instead of a cost-benefit standard. As consumer demand for “green” products continues to increase, green chemistry and green chemical engineering are emerging as significant stakeholders in the realm of environmental management of childhood asthma.

Efforts to affect policy issues at the federal level have been led by many nonprofit health and advocacy organizations such as the ALA (2009), the Natural Resources Defense Council (Ginty 2015), the American Public Health Association (APHA 2001), the AAP (Lara et al. 2002), and others. These organizations work to educate legislators and advocate for policy change related to many factors (e.g., secondhand smoke, indoor air quality, climate change, energy use, just treatment, access to care) that affect health outcomes in children. They recognize that the issues resonate with groups (e.g., groups’ members, parents, teachers, and health-affected individuals) that they would like to engage as advocates for effective public health policies. They have also formed coalitions with others to increase their power to affect change at the policy level.

For decades, these same organizations have advocated for better diagnosis, prevention, management, and just treatment of individuals and populations disproportionately affected with asthma, and they have led efforts to build awareness of the social justice issues associated with poor air quality. The social determinants of health interconnect with these aims and impact the outcomes. Approximately 13.4% of non-Hispanic black children in the United States had asthma in 2014 (CDC 2014b), the highest rate among racial groups. Some of this disparity is undoubtedly due to disproportionate exposure to air pollutants such as particle pollution. A report, “Coal Blooded: Putting Profits Before People,*”* released in 2012 by the National Association for the Advancement of Colored People (NAACP), Indigenous Environmental Network, and the Little Village Environmental Justice Organization (NAACP 2012), highlights the fact that coal-fired power plants are disproportionately sited in communities of color and communities of lower income. Residential proximity to coal power plants is associated with increased likelihood of hospitalizations for asthma and other respiratory diseases (Liu et al. 2012).

The face of the United States has been changing rapidly. A 2014 U.S. Census Bureau report found that the majority of U.S. children < 5 years old are nonwhite (Colby and Ortman 2014). Hispanic children represent one of the fastest growing racial/ethnic groups. In 2014, Hispanic children represented 24.4% of those < 18 years old, but they are projected to represent 33.5% by the year 2060 (Colby and Ortman 2014). The families of this growing population of Hispanic children, and the families of African-American children burdened with asthma and whose voices have often been marginalized, will have greater influence on the issue in the decades to come.

In the United States, a generation of influential and long-standing change agents including Benjamin Chavis Muhammad, Robert Bullard, Peggy Shepard, Beverly Wright, and Tom Goldtooth, as well as organizations such as the United Church of Christ’s Commission on Racial Justice and the West Harlem Environmental Action, Inc. (WE ACT for Environmental Justice) have led the environmental justice movement for decades. In addition, a new influential voice, GreenLatinos is gaining momentum. As a national nonprofit organization, GreenLatinos has assembled a broad coalition of Latino leaders who are committed to addressing environmental issues that affect the health and welfare of their communities and their children. Relatively newer voices have emerged from online communities and the world of blogging in the last decade that have, and continue to become, influential in efforts to raise awareness about asthma prevention, care, and just treatment. Besides blogs managed by nonprofit advocacy organizations, “Mom groups,” such as Mom’s Clean Air Force and MomsRising, are calling attention on their blogs to the justice and air quality issues that contribute to asthma exacerbations and amassing large followings. In addition to raising awareness, blogs rank high with consumers for trust, popularity, and influence (Technorati 2013). A new term—“Eco-Moms”—has been coined for the environmental purchasing power of these influential moms. One marketing research company, EcoFocus Worldwide, estimated in a 2010 report that the Eco-Moms market, which includes more than 50 million women and represents 69% of all moms, holds more than $1.45 trillion in buying power (EcoFocus 2010). These moms can move markets by demanding products and services that are safer for their families, and this power can transform into political influence. This power was demonstrated when industry voluntarily removed baby bottles that contained bisphenol A from store shelves in response to market pressure from moms and other advocates, all before the U.S. Food and Drug Administration enacted a 2012 ban on the use of the additive in baby bottles.

This article provides a very broad overview of some of the key players that influence the environmental contributions to childhood asthma. It should suggest to the reader that with regard to CEH, the players and issues are many and complex. The need for a comprehensive stakeholder management tool (map) that identifies the stakeholders in CEH, their respective interests and roles, and how they are interconnected, is warranted for effective systems change efforts. It would inform future initiatives borne of the recommendations identified by “A Blueprint for Protecting Children’s Environmental Health: An Urgent Call to Action” (CEHN 2015a). The current burden of childhood asthma and other health and developmental conditions point to the need for the assessment and enhancement of measurable, evidence-based CEH indicators (both environmental indicators and increased biomonitoring efforts) that can make the case for protective decisions and actions and demonstrate progress and accountability. A CEH collective and coordinating entity would be devoted to establishing a connected and vibrant children’s environmental health community. We propose that such a collective address the following goals:

Mobilize society to take action on children’s environmental health.
Place a strong priority on children and families.
Create the knowledge that is essential for effective action and make better use of the knowledge that is already available.
Help to marshal the engine of the economy to achieve environments in which children can thrive and enjoy abundant opportunities for building a sustainable, economically secure future.
Build the political will in our institutions of government for child-centered policies.

If we hope to address current and future CEH concerns, we will need to find new and creative solutions that consider and connect all the diverse homes of influence and encourage cross-sector collaboration that consistently places the child at the center.
